# Development and validation of a machine learning model for colorectal cancer status classification using NHANES data: A cross-sectional study

**DOI:** 10.1097/MD.0000000000049114

**Published:** 2026-06-05

**Authors:** Jialong Chen

**Affiliations:** a Department of Medical Imaging, Lianjiang County General Hospital, Fuzhou, China.

**Keywords:** colorectal cancer, explainable artificial intelligence, machine learning, NHANES, SHAP, status classification

## Abstract

Colorectal cancer (CRC) is a leading cause of cancer-related morbidity and mortality worldwide. Tools based on routinely collected variables may help identify prevalent CRC status and support clinical evaluation. Traditional approaches often rely on limited predictors and may not capture the multidimensional nature of CRC. Data were obtained from the National Health and Nutrition Examination Survey 1999–2018. Among 53,881 participants, 420 reported physician-diagnosed CRC (MCQ220). A 1:10 stratified case-control sample was constructed (420 cases, 4200 controls) and randomly split into training (70%) and internal validation (30%) sets. Missing data were imputed separately in the training and validation sets using a random forest–based method. SMOTE was applied only to the training set. Logistic regression, random forest, support vector machine, k-nearest neighbors, and extreme gradient boosting (XGBoost) were compared. Performance was primarily assessed by discrimination in the held-out validation set. For the final XGBoost model, exploratory post hoc probability calibration analyses were performed on validation-set predictions using raw probabilities, prior prevalence correction, and Platt scaling. Model interpretability was examined using Shapley Additive Explanations (SHAP), and a web-based CRC status classifier was developed. XGBoost showed the best discrimination in the validation cohort, with an area under the receiver operating characteristic curve of 0.787 (95% confidence interval 0.749–0.825). At the Youden-index cutoff, sensitivity was 77.0%, specificity 67.6%, PPV 19.2%, and NPV 96.7%. In exploratory probability-based analyses, decision curve analysis using Platt-scaled probabilities showed greater net benefit than treat-all and treat-none strategies across low-to-moderate threshold probabilities. post hoc calibration analyses fitted and assessed on the validation-set predictions showed improved apparent agreement after Platt scaling, with a Brier score of 0.191 and a Hosmer–Lemeshow *P* value of 0.086. SHAP identified key predictors, including alcohol use, hypertension, age, triglycerides, absolute lymphocyte count, blood lead, serum cotinine, and neutrophil-to-lymphocyte ratio. An interpretable machine learning framework integrating multidomain predictors enabled effective CRC status classification in a large population-based cohort. Discrimination was strong, whereas probability-based outputs after post hoc calibration should be considered exploratory pending independent confirmation. The model may support clinical evaluation and triage for individuals requiring further assessment.

## 1. Introduction

Colorectal cancer (CRC) is among the most commonly diagnosed malignancies worldwide and remains a leading cause of cancer-related mortality despite advances in screening and treatment strategies.^[1–3]^ Globally, CRC imposes a substantial public health burden, with incidence and mortality rates continuing to rise in many regions, particularly among aging populations.^[4,5]^ This study uses a cross-sectional design to classify colorectal cancer status at the time of examination. Unlike prospective models that predict future cancer development, our classifier identifies individuals who already have CRC based on routinely available clinical measurements.^[6]^

A potential application of such a classifier is to complement existing clinical pathways by helping to flag individuals who may benefit from further evaluation rather than serving as a standalone screening tool.^[7–9]^ While these factors are clinically meaningful, they fail to fully capture the multifactorial nature of colorectal carcinogenesis, which involves complex interactions among demographic characteristics, lifestyle exposures, metabolic dysregulation, systemic inflammation, and environmental toxicants.^[10–13]^ As a result, existing approaches often show limited discrimination and insufficient individual-level risk stratification.^[14]^

Large population-based datasets offer new opportunities to develop more comprehensive CRC status classification models. The National Health and Nutrition Examination Survey (NHANES) is a nationally representative program that integrates detailed demographic information, questionnaire data, laboratory biomarkers, and measurements of environmental exposures.^[15,16]^ Previous studies have successfully used NHANES data to investigate associations between lifestyle factors, metabolic abnormalities, inflammatory markers, and cancer risk.^[17–20]^ However, the high dimensionality of NHANES variables, the presence of nonlinear relationships, and pronounced class imbalance between CRC cases and controls pose significant challenges for traditional regression-based modeling approaches.^[9,21]^

Machine learning (ML) methods have emerged as powerful tools for handling complex, high-dimensional epidemiological data in status classification. Algorithms such as logistic regression extensions, random forest, support vector machines, KNN, and gradient boosting can model nonlinear associations and higher-order interactions without requiring strong parametric assumptions.^[9,11,22,23]^ Recent studies have demonstrated that ML-based models may outperform conventional statistical methods in CRC status classification and related oncological applications.^[9,24–26]^ Nevertheless, concerns regarding model interpretability, overfitting, and limited assessment of calibration and clinical utility have restricted their broader adoption in clinical and public health settings.^[9,21,27]^

To address the interpretability challenge, explainable artificial intelligence techniques have been increasingly incorporated into ML-based health research. Shapley Additive Explanations (SHAP) provide a unified and theoretically grounded framework to quantify the contribution of individual features to model predictions at both the global and individual levels.^[26,28,29]^ By enabling transparent feature attribution, SHAP facilitates feature prioritization, model simplification, and clinical interpretability, which are essential for translating ML models into real-world decision-making tools.^[30,31]^

In this study, we utilized NHANES data from 1999 to 2018 to develop and internally validate a machine learning framework for CRC status classification. Multiple algorithms were compared, and model performance was assessed in terms of discrimination, calibration, and decision-analytic utility. Model interpretability was examined using SHAP-based global explanations. Finally, the validated model was deployed as a web-based CRC status classifier to facilitate individualized status estimation in population-based settings.

## 2. Methods

### 2.1. Data source and study population

Data were obtained from the NHANES, a nationally representative cross-sectional survey conducted by the Centers for Disease Control and Prevention. In this study, multiple continuous NHANES cycles from 1999 to 2018 were combined to ensure adequate sample size and statistical power.

Participants with complete questionnaire data regarding CRC status were eligible for inclusion. CRC status was determined based on self-reported physician diagnosis from standardized NHANES questionnaire items. Although mortality data were available through NHANES-linked files, mortality outcomes were not included in the present analysis. Individuals with missing outcome information were excluded.

The study population was divided into a CRC group and a non-CRC control group. CRC was treated as a binary outcome, coded as 1 for CRC cases and 0 for non-CRC participants. NHANES is a publicly available, de-identified dataset collected by the U.S. Centers for Disease Control and Prevention National Center for Health Statistics. All NHANES protocols were approved by the National Center for Health Statistics Research Ethics Review Board (Protocol #98-12, with continued review waivers since 2004). Because we analyzed only publicly available, de-identified data, no additional institutional review board approval was required for this secondary analysis.

### 2.2. Candidate variables and feature preprocessing

Guided by prior literature and considering the clinical availability of routinely collected indicators, a total of 28 candidate variables were initially considered for model development. These variables covered multiple clinically relevant domains, including demographic characteristics (such as age, sex, race/ethnicity, and poverty–income ratio), lifestyle factors (including smoking and alcohol consumption), inflammatory and hematological markers, metabolic indicators, as well as environmental and chemical exposure biomarkers.

Continuous variables were retained in their original scales, while categorical variables were encoded according to NHANES standard definitions. A complete list of candidate predictors, NHANES variable codes, measurement units, and any transformations is provided in Supplementary Table S1.

### 2.3. Construction of the analytic cohort and handling of class imbalance

**Figure 1.**
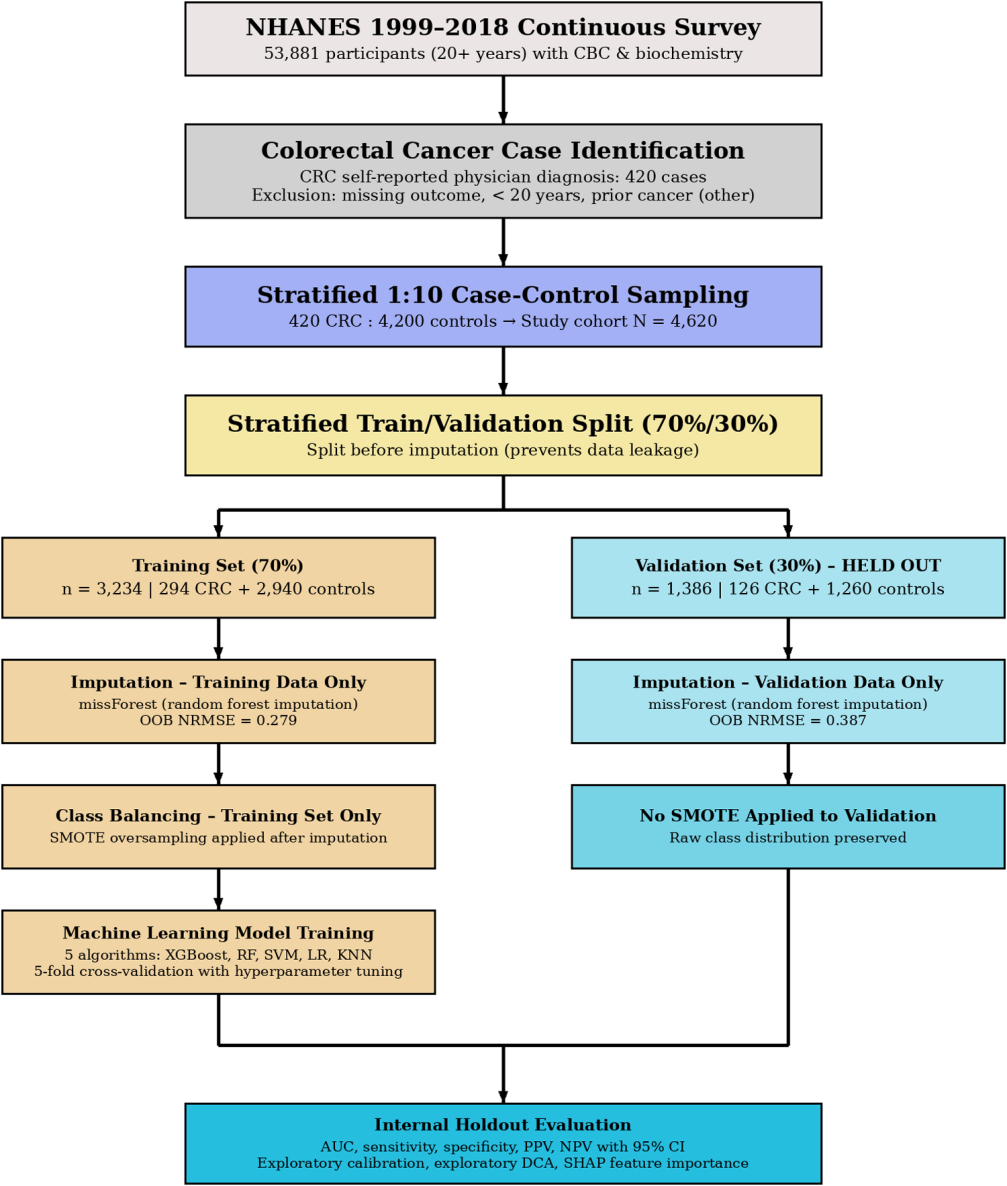
Study design and analytical pipeline. Participants were drawn from NHANES 1999–2018. A 1:10 stratified case-control sample was constructed (420 CRC cases: 4200 controls). The cohort was split into training (70%, n = 3234) and validation (30%, n = 1386) sets before imputation to prevent data leakage. missForest imputation was performed independently on each set (Training OOB NRMSE = 0.279; Validation OOB NRMSE = 0.387). SMOTE was applied only to the training set. The validation set was left untouched throughout the entire training process.

The overall study design and analytical pipeline are shown in Figure 1. CRC was rare in the full NHANES sample, a 1:10 stratified case-control analytic cohort was constructed for model development and internal validation (420 CRC cases and 4200 controls; n = 4620). To prevent data leakage, the analytic cohort was randomly split into a training set (70%, n = 3234) and an independent validation set (30%, n = 1386; 126 CRC cases and 1260 controls) prior to imputation.

Missing values were imputed using a random forest–based method (missForest). Imputation was performed separately within the training set and the validation set. To address class imbalance during model training, SMOTE oversampling was applied to the training set only after imputation. The validation set was not oversampled and retained the original 1:10 case-control ratio.

### 2.4. Model Development and Comparison

Multiple machine learning algorithms were evaluated to identify the optimal model for CRC status classification, including:

- Logistic regression (LR),
- Random forest (RF),
- Extreme gradient boosting (XGBoost),
- Support vector machine (SVM), and
- K-nearest neighbors (KNN) classifier.

All models were trained using the same training cohort and identical feature sets to ensure fair comparison. Hyperparameters were tuned using internal cross-validation within the training cohort. Model performance was primarily assessed using the area under the receiver operating characteristic curve (AUC). Hyperparameter optimization was performed for each algorithm using 5-fold stratified cross-validation within the training set. The validation set was not used for model training or hyperparameter selection; it was reserved for final performance evaluation and post hoc probability calibration analyses. Search grids and optimal values were as follows: XGBoost max_depth ∈ {3, 5, 7}, learning rate (eta) ∈ {0.01, 0.05, 0.1}, number of rounds determined by early stopping (optimal: max_depth = 3, eta = 0.1, nrounds = 55); Random Forest — mtry ∈ {2, 5, 8, √p} (optimal: mtry = 8); SVM — sigma ∈ {0.01, 0.1}, C ∈ {0.1, 1, 10} (optimal: sigma = 0.01, C = 0.1); KNN — k ∈ {5, 7, 9, 11, 13} (optimal: k = 11); Logistic Regression — no tuning required. Among the evaluated models, XGBoost demonstrated the best discriminative performance and was therefore selected as the final model for calibration, SHAP analysis, decision curve analysis, and web-based deployment.

### 2.5. Probability calibration

Because the 1:10 case-control sampling increases the apparent CRC prevalence in the analytic cohort relative to the full NHANES population, raw model probabilities are not directly interpretable as population-level probabilities. Therefore, an exploratory post hoc probability calibration analysis was performed for the final XGBoost model after primary discrimination assessment on the held-out validation set. We compared: raw predicted probabilities, a prior prevalence-correction approach, and Platt scaling. Platt scaling fits a logistic regression model to map the model’s score to calibrated probabilities. In this study, Platt scaling was fitted on the validation-set predictions to examine whether model outputs could be better aligned with observed CRC proportions under the case-control sampling scheme. Accordingly, calibration plots, the Brier score, the Hosmer–Lemeshow goodness-of-fit test, and decision curve analysis based on calibrated probabilities were treated as exploratory secondary analyses rather than fully independent confirmatory estimates.

### 2.6. Model evaluation and validation

Model discrimination was evaluated using receiver operating characteristic (ROC) curves and the AUC with 95% confidence intervals. Classification metrics included sensitivity, specificity, accuracy, positive predictive value (PPV), and negative predictive value (NPV), computed at the optimal cutoff determined by the Youden index for each model.

Clinical utility was evaluated using decision curve analysis for the final XGBoost model, comparing net benefit against treat-all and treat-none strategies across a clinically relevant range of low-to-moderate threshold probabilities. Calibration was evaluated as described above.

### 2.7. Model interpretability

Global model interpretability for the final XGBoost model was examined using SHapley Additive exPlanations (SHAP). Feature importance was summarized by mean absolute SHAP values and visualized using SHAP summary plots (bar and beeswarm) on the validation cohort.

### 2.8. Development of a web-based CRC status classifier

A web-based CRC status classifier was developed using the Shiny framework. The application outputs an estimated probability of prevalent CRC status based on user-entered predictors from the final XGBoost model and is intended for investigational use only (not for clinical decision-making). The online application is accessible at: https://maghaz.shinyapps.io/crc_shiny_calculator/.

### 2.9. Statistical analysis

All analyses were conducted using R software (version 4.1.1). Machine learning models were implemented using established R packages, including xgboost, randomForest, and e1071. Statistical significance was assessed at a 2-sided *P* value < 0.05 where applicable.

## 3. Results

### 3.1. Baseline characteristics of the study population

Baseline characteristics of the overall NHANES study population (n = 53,881; CRC = 420) are summarized in Table 1. Compared with controls, participants with self-reported CRC were older and more frequently reported diabetes and hypertension. Several inflammatory composite indices were higher in the CRC group, including neutrophil-to-lymphocyte ratio (NLR), platelet-to-lymphocyte ratio, and systemic immune-inflammation index. CRC participants also showed differences in selected cardiometabolic and environmental exposure markers, including higher fasting plasma glucose and HbA1c, lower total cholesterol, and higher blood lead concentration. No clear between-group differences were observed for multiple other variables (e.g., waist circumference, serum cotinine, cadmium, mercury, CRP, white blood cell count, absolute neutrophil count, platelet count, triglycerides, and HDL cholesterol; Table 1).

**Table 1:** Baseline characteristics of the overall NHANES study population.

	level	Overall	Control	CRC	p	Missing (%)
n		53,881	53,461	420		
Age		47.45 ± 19.14	47.39 ± 19.06	55.67 ± 26.89	<0.001	0.00%
Gender (%)	Male	25,864 (48.0)	25,656 (48.0)	208 (49.5)	0.564	0.00%
	Female	28,017 (52.0)	27,805 (52.0)	212 (50.5)		
Race (%)	Mexican American	11,040 (20.5)	10,974 (20.5)	66 (15.7)	<0.001	0.00%
	Other Hispanic	3185 (5.9)	3169 (5.9)	16 (3.8)		
	Non-Hispanic White	21,589 (40.1)	21,368 (40.0)	221 (52.6)		
	Non-Hispanic Black	12,993 (24.1)	12,907 (24.1)	86 (20.5)		
	Other Race	5074 (9.4)	5043 (9.4)	31 (7.4)		
Education (%)	<9th Grade	8479 (15.9)	8421 (15.9)	58 (16.5)	0.814	0.40%
	9-11th Grade	8489 (16.0)	8435 (16.0)	54 (15.4)		
	High School	13,577 (25.5)	13,495 (25.5)	82 (23.4)		
	Some College	14,182 (26.7)	14,080 (26.7)	102 (29.1)		
	College Grad	8457 (15.9)	8402 (15.9)	55 (15.7)		
Poverty income ratio		2.43 ± 1.67	2.44 ± 1.67	2.32 ± 1.59	0.183	9.70%
BMI		28.24 ± 6.71	28.25 ± 6.71	27.52 ± 7.14	0.038	2.10%
WAIST		96.29 ± 16.39	96.30 ± 16.35	96.13 ± 21.31	0.86	3.50%
Smoker_Bio (%)	No	35,583 (73.4)	35,318 (73.3)	265 (79.1)	0.021	6.80%
	Yes	12,906 (26.6)	12,836 (26.7)	70 (20.9)		
Drinker (%)	No	7496 (29.4)	7388 (29.3)	108 (34.1)	0.073	0.20%
	Yes	18,034 (70.6)	17,825 (70.7)	209 (65.9)		
Diabetes (%)	No	48,170 (89.4)	47,857 (89.5)	313 (74.5)	<0.001	0.00%
	Yes	5709 (10.6)	5602 (10.5)	107 (25.5)		
Hypertension (%)	No	36,850 (68.7)	36,710 (69.0)	140 (33.3)	<0.001	0.40%
	Yes	16,801 (31.3)	16,521 (31.0)	280 (66.7)		
Serum Cotinine (ng/mL)		60.41 ± 130.69	60.47 ± 130.68	52.22 ± 131.86	0.25	0.00%
Blood Cadmium (µg/L)		0.52 ± 0.59	0.52 ± 0.59	0.56 ± 0.43	0.324	0.00%
Blood Lead (µg/dL)		2.49 ± 2.70	2.49 ± 2.70	2.86 ± 2.46	0.012	0.00%
Blood Mercury (µg/L)		1.59 ± 2.59	1.59 ± 2.59	1.66 ± 2.49	0.678	0.00%
Neutrophil-to-Lymphocyte ratio (NLR)		2.16 ± 1.19	2.16 ± 1.19	2.74 ± 1.52	<0.001	0.30%
Platelet-to-lymphocyte ratio (PLR)		126.71 ± 49.58	126.60 ± 49.39	142.43 ± 69.10	<0.001	0.30%
Systemic immune-inflammation index (SII)		539.36 ± 377.89	538.54 ± 377.27	651.55 ± 440.72	<0.001	0.40%
Serum C-reactive protein (mg/L)		0.46 ± 0.83	0.46 ± 0.83	0.55 ± 1.04	0.125	0.30%
White blood cell count (10^3^ cells/µL)		7.25 ± 2.33	7.26 ± 2.33	7.11 ± 2.35	0.241	0.20%
Absolute neutrophil count (10^3^ cells/µL)		4.29 ± 1.82	4.29 ± 1.82	4.29 ± 1.64	0.999	0.30%
Absolute lymphocyte count (10^3^ cells/µL)		2.22 ± 0.97	2.22 ± 0.97	2.13 ± 1.34	0.097	0.30%
Platelet count (10^3^ cells/µL)		257.33 ± 69.27	257.33 ± 69.14	256.64 ± 85.62	0.852	0.20%
Fasting plasma glucose (mg/dL)		110.35 ± 43.18	110.29 ± 43.13	119.07 ± 49.01	0.006	48.00%
Glycated hemoglobin (HbA1c, %)		5.66 ± 1.10	5.66 ± 1.10	5.95 ± 1.25	<0.001	0.40%
Total cholesterol (mg/dL)		196.84 ± 43.41	196.89 ± 43.38	189.16 ± 46.81	0.001	3.40%
Triglycerides (mg/dL)		135.81 ± 114.19	135.81 ± 114.30	136.68 ± 96.07	0.911	48.00%
High-density lipoprotein cholesterol (mg/dL)		52.43 ± 15.89	52.43 ± 15.87	52.44 ± 17.45	0.995	3.40%

Data are presented as mean ± standard deviation or n (%). Missingness is shown for the overall NHANES sample before analytic cohort construction. Percentages may not sum to 100% because of rounding.

BMI = body mass index, CRC = colorectal cancer, HbA1c = glycated hemoglobin, HDL = high-density lipoprotein, NLR = neutrophil-to-lymphocyte ratio, PLR = platelet-to-lymphocyte ratio, SII = systemic immune-inflammation index.

### 3.2. Comparison of machine learning models in the validation cohort

**Figure 2.**
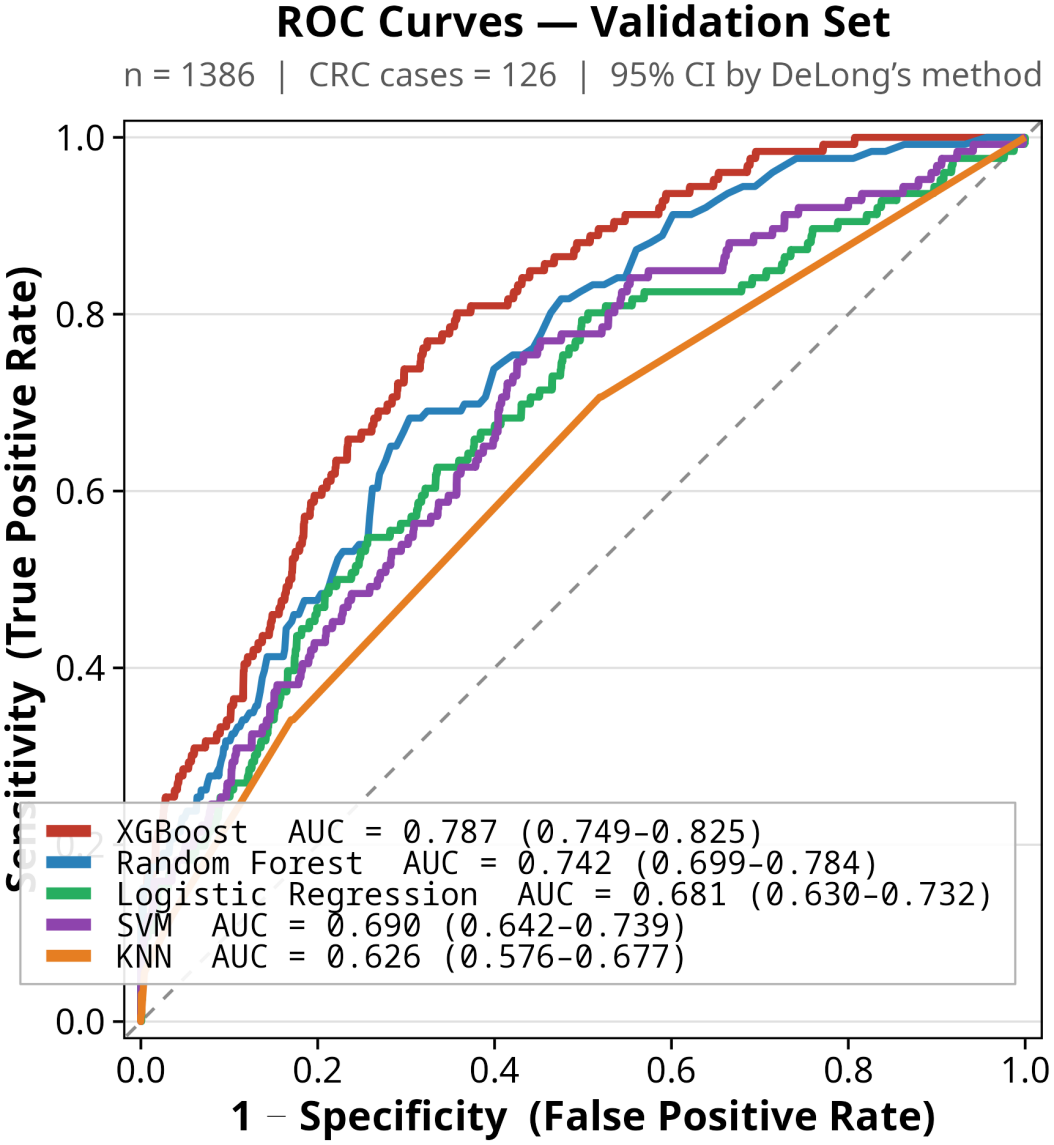
Performance comparison of machine learning models. 95% confidence intervals computed using DeLong’s method.

Five machine learning models LR, RF, SVM, KNN, and extreme gradient boosting (XGBoost) were compared in the validation cohort. ROC curves are shown in Figure 2, and detailed performance metrics are summarized in Tables 2 and 3.

**Table 2:** Validation-set discrimination and classification performance of machine learning models.

Model	AUC (95% CI)	Threshold	Sensitivity (95% CI)	Specificity (95% CI)	PPV (95% CI)	NPV (95% CI)	Accuracy (95% CI)
XGB	0.787 (0.749-0.825)	0.0738	0.770 (0.689-0.835)	0.676 (0.650-0.701)	0.192 (0.160-0.229)	0.967 (0.953-0.977)	0.685 (0.660-0.709)
RF	0.742 (0.699-0.784)	0.107	0.683 (0.597-0.757)	0.696 (0.670-0.721)	0.183 (0.151-0.221)	0.956 (0.941-0.968)	0.695 (0.670-0.718)
SVM	0.690 (0.642-0.739)	0.0745	0.754 (0.672-0.821)	0.567 (0.540-0.595)	0.148 (0.123-0.178)	0.958 (0.942-0.971)	0.584 (0.558-0.610)
LR	0.681 (0.630-0.732)	0.0692	0.802 (0.723-0.862)	0.494 (0.467-0.522)	0.137 (0.114-0.164)	0.961 (0.944-0.974)	0.522 (0.496-0.549)
KNN	0.626 (0.576-0.677)	0.0871	0.706 (0.622-0.779)	0.481 (0.453-0.509)	0.120 (0.098-0.145)	0.942 (0.922-0.958)	0.501 (0.475-0.528)

Thresholds were selected using the Youden index.

AUC = area under the receiver operating characteristic curve, CI = confidence interval, KNN = k-nearest neighbors, LR = logistic regression, NPV = negative predictive value, PPV = positive predictive value, RF = random forest, SVM = support vector machine, XGB = XGBoost.

**Table 3:** Expanded validation-set performance metrics of machine learning models.

Model	AUC (95% CI)	Threshold	Sensitivity (95% CI)	Specificity (95% CI)	Accuracy (95% CI)	PPV (95% CI)	NPV (95% CI)	F1
XGB	0.787 (0.749–0.825)	0.073842	0.770 (0.689–0.835)	0.676 (0.650–0.701)	0.685 (0.660–0.709)	0.192 (0.160–0.229)	0.967 (0.953–0.977)	0.307
RF	0.742 (0.699–0.784)	0.107	0.683 (0.597–0.757)	0.696 (0.670–0.721)	0.695 (0.670–0.718)	0.183 (0.151–0.221)	0.956 (0.941–0.968)	0.289
SVM	0.690 (0.642–0.739)	0.074464	0.754 (0.672–0.821)	0.567 (0.540–0.595)	0.584 (0.558–0.610)	0.148 (0.123–0.178)	0.958 (0.942–0.971)	0.248
LR	0.681 (0.630–0.732)	0.069245	0.802 (0.723–0.862)	0.494 (0.467–0.522)	0.522 (0.496–0.549)	0.137 (0.114–0.164)	0.961 (0.944–0.974)	0.234
KNN	0.626 (0.576–0.677)	0.087121	0.706 (0.622–0.779)	0.481 (0.453–0.509)	0.501 (0.475–0.528)	0.120 (0.098–0.145)	0.942 (0.922–0.958)	0.205

AUC = area under the receiver operating characteristic curve, CI = confidence interval, KNN = k-nearest neighbors, LR = logistic regression, NPV = negative predictive value, PPV = positive predictive value, RF = random forest, SVM = support vector machine, XGB = XGBoost.

Among the 5 models, XGBoost achieved the best discrimination, with an AUC of 0.787 (95% confidence interval [CI]: 0.749–0.825). RF ranked second (AUC 0.742, 95% CI: 0.699–0.784), followed by SVM (AUC 0.690, 95% CI: 0.642–0.739) and LR (AUC 0.681, 95% CI: 0.630–0.732), whereas KNN showed the lowest AUC (0.626, 95% CI: 0.576–0.677) (Table 2; Fig. 2). Using model-specific Youden-index thresholds, XGBoost achieved a sensitivity of 0.770 (95% CI: 0.689–0.835) and specificity of 0.676 (95% CI: 0.650–0.701), with PPV 0.192 (95% CI: 0.160–0.229) and NPV 0.967 (95% CI: 0.953–0.977) (Table 2). Corresponding classification metrics for the other models are provided in Tables 2 and 3.

### 3.3. Decision curve analysis

**Figure 3.**
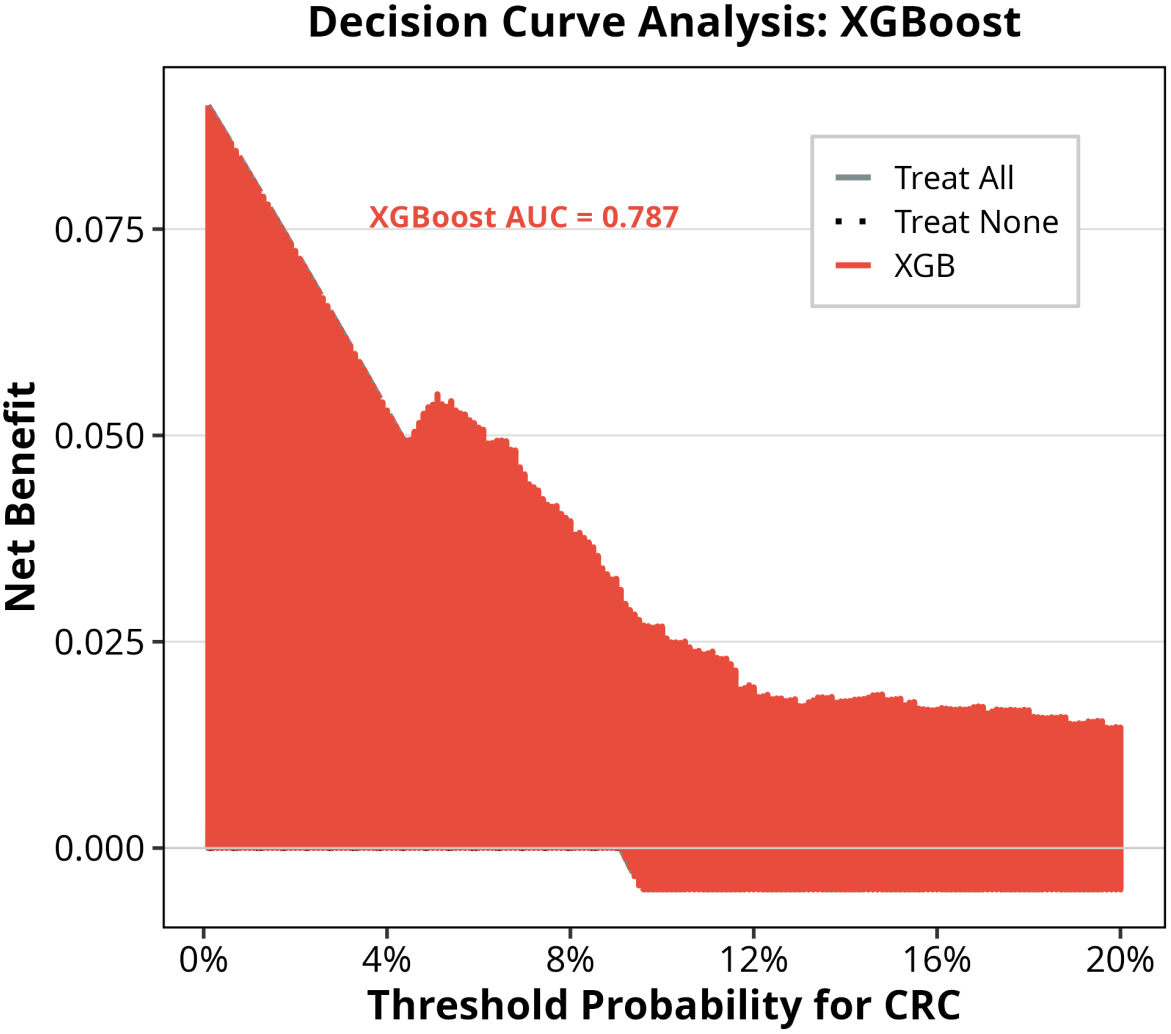
Decision curve analysis for XGBoost on the validation set (n = 1386). Probabilities are Platt-scaled using a post hoc calibration model fitted on the validation-set predictions. Net benefit is plotted across a range of threshold probabilities. XGBoost (solid line) provides apparent positive net benefit relative to treat-all and treat-none strategies across threshold probabilities of approximately 0.5%–15%; these results should be interpreted as exploratory.

Decision curve analysis for the final XGBoost model is presented in Figure 3. The XGBoost model showed greater net benefit than the treat-all and treat-none strategies across a clinically relevant range of low-to-moderate threshold probabilities, supporting potential utility in population evaluation settings.

### 3.4. Calibration performance

**Figure 4.**
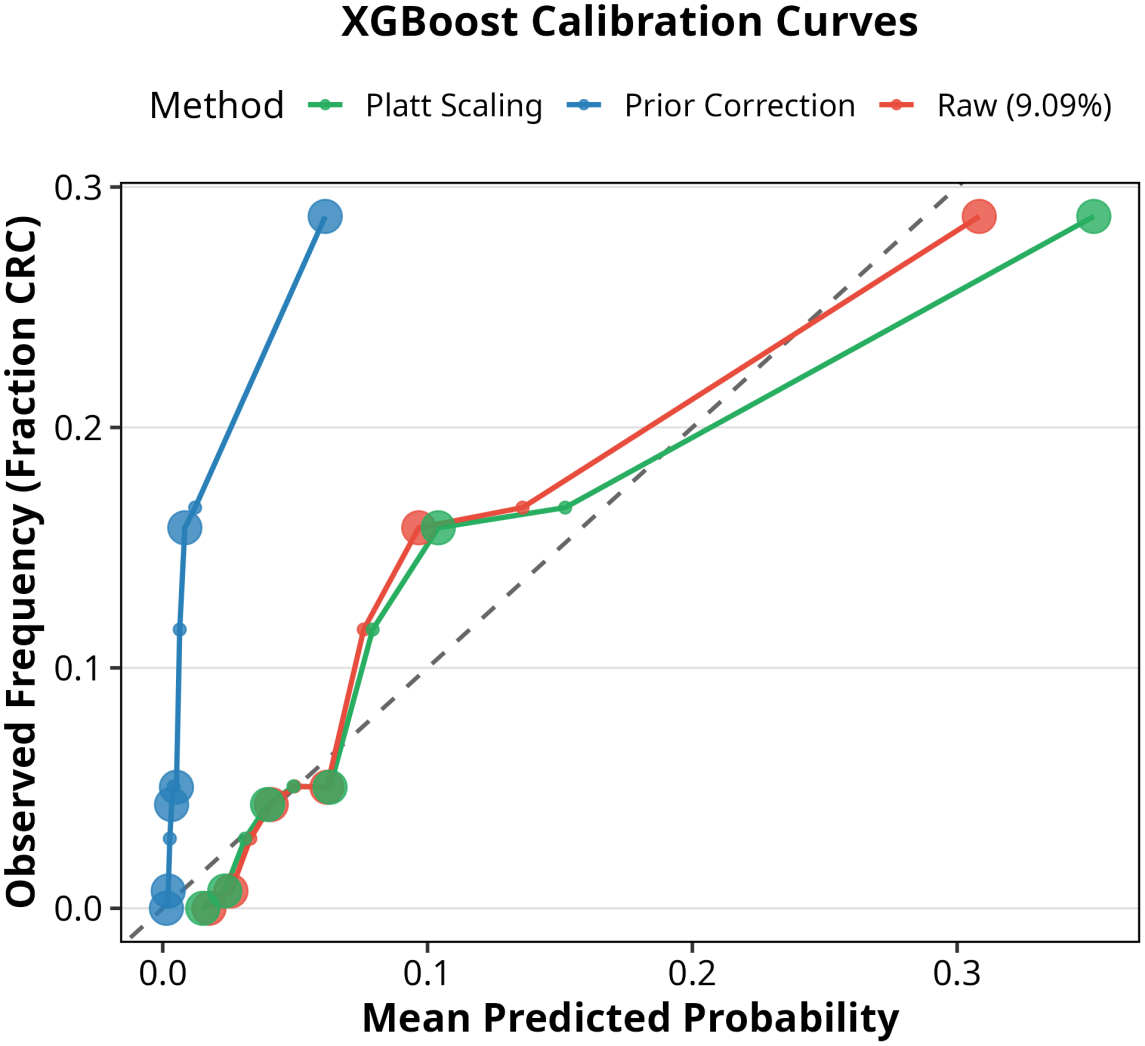
Calibration comparison: prior correction versus Platt scaling. Predicted probabilities (x-axis) versus observed CRC proportions (y-axis) in 10 equal-width bins. Platt-scaled probabilities (orange) are more closely aligned with observed CRC proportions than prior-corrected probabilities (blue), with Hosmer-Lemeshow test results of *P* = .086 for Platt scaling and *P* < .001 for prior correction. Because Platt scaling was fitted and assessed in the same validation set, this figure represents a post hoc exploratory calibration analysis. The Brier score was computed using the same Platt-scaled predictions.

Calibration results for XGBoost are shown in Figure 4. The raw predicted probabilities (reflecting the enriched 1:10 case-control sampling) and a prior prevalence-correction approach exhibited visible miscalibration. In contrast, in this post hoc exploratory analysis, Platt scaling improved apparent agreement between predicted probabilities and observed CRC proportions across most bins. After Platt scaling, the model achieved a Brier score of 0.191 and an acceptable Hosmer–Lemeshow goodness-of-fit result (*P* = .086), indicating reasonable apparent probabilistic calibration (Fig. 4). Because Platt scaling was fitted and assessed on the same held-out validation set, these calibration results should be interpreted cautiously as exploratory rather than confirmatory.

### 3.5. Model interpretability using SHAP

**Figure 5.**
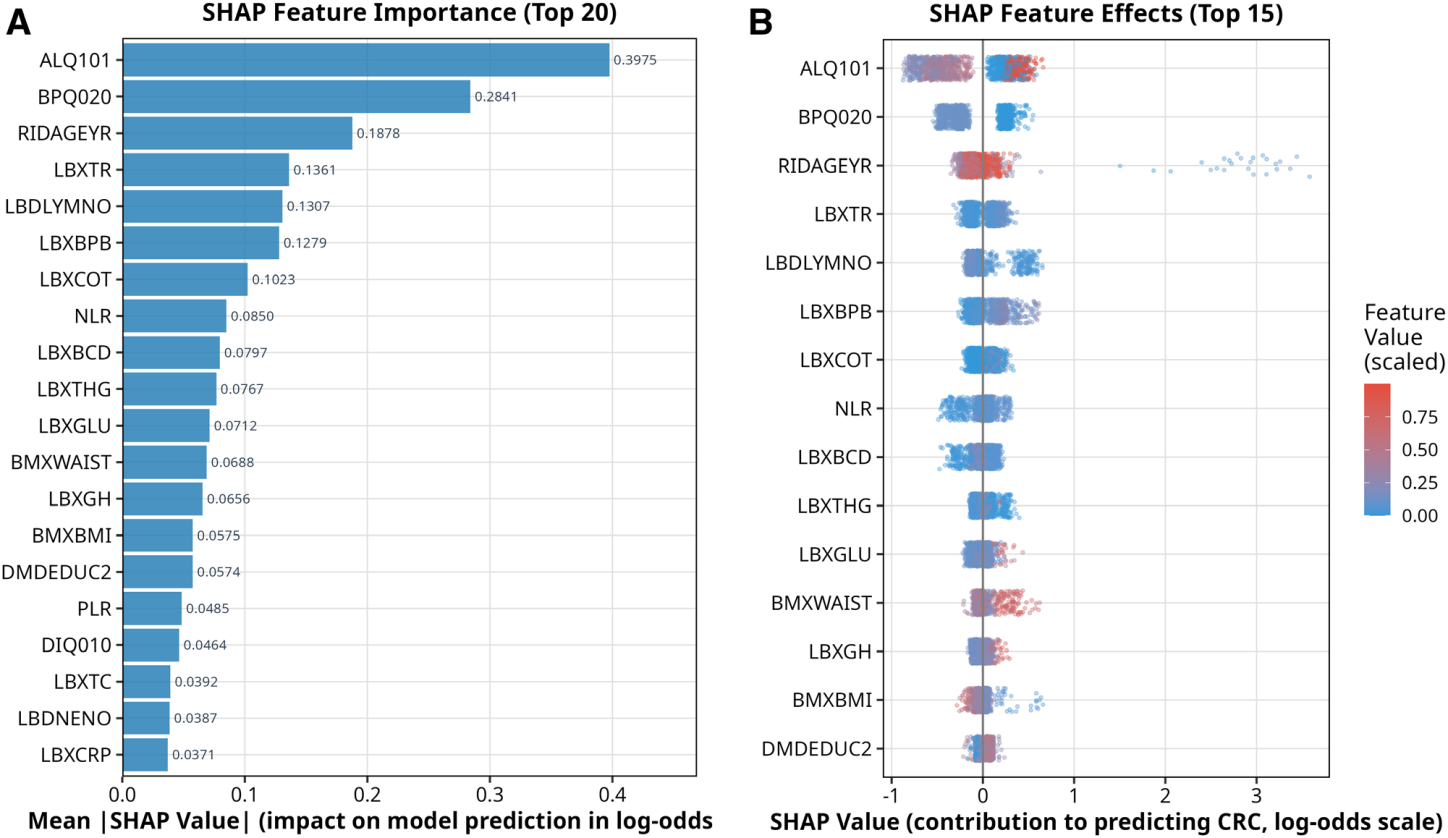
SHAP (SHapley Additive exPlanations) feature importance for XGBoost on the validation set. INVESTIGATIONAL USE ONLY. Baseline predicted probability = 6.97% (expected model output in the validation cohort under the case-control sampling scheme). (A) Mean absolute SHAP values indicate feature importance. (B) Beeswarm plot shows the direction and magnitude of each feature’s contribution per sample. Positive SHAP values increase the predicted probability above the 6.97% baseline; negative SHAP values decrease it.

**Figure 6.**
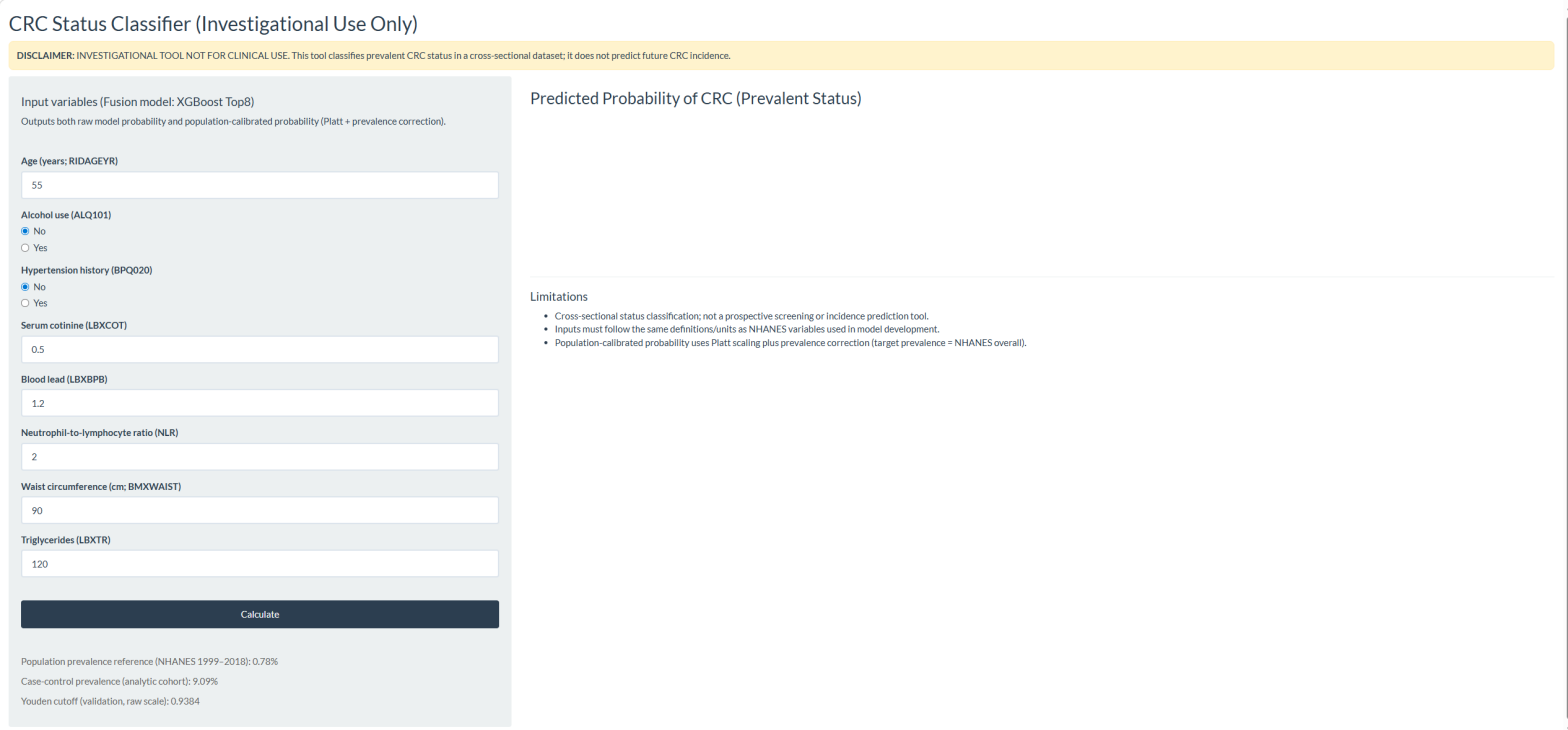
Web-based CRC status classifier interface (investigational use only). Screenshot of the Shiny application developed from the final XGBoost model to estimate the probability of prevalent colorectal cancer (CRC) status using user-entered predictor values. The interface displays model outputs and key references, including the NHANES population prevalence (0.78%), and includes a prominent disclaimer indicating that the tool is investigational and not intended for clinical use.

Global interpretability of the final XGBoost model was assessed using SHAP on the validation cohort (Figs. 5A–B). The SHAP feature-importance ranking indicated that predictive contributions were concentrated in a subset of variables. The top contributors included alcohol use status (ALQ101), self-reported hypertension (BPQ020), age (RIDAGEYR), triglycerides (LBXTR), absolute lymphocyte count (LBDLYMNO), blood lead (LBXBPB), serum cotinine (LBXCOT), and NLR (Fig. 5A). The beeswarm plot further illustrated the distribution and direction of feature effects across individuals (Fig. 5B). SHAP values quantify how individual features shift the model output from the baseline predicted probability (6.97%) toward higher or lower probabilities of prevalent CRC status. The web-based CRC status classifier interface is shown in Figure 6. The application was developed from the final XGBoost model for investigational use only and displays calibrated probability estimates based on user-entered predictor values.

## 4. Discussion

This model classifies prevalent CRC status at the time of NHANES examination. It identifies individuals who already have CRC based on cross-sectional clinical data and cannot be used to predict future cancer development. A potential application is as a complement to existing clinical evaluation pathways for individuals presenting with relevant clinical findings, rather than as a population screening test.^[22,32–34]^

CRC is a multifactorial disease shaped by cumulative contributions from aging, lifestyle factors, cardiometabolic dysfunction, immune–inflammatory activation, and exogenous exposures.^[23,35–37]^ Consistent with this framework, SHAP-based global explanations indicated that predictive contributions were concentrated in a subset of routinely available variables, supporting parsimonious interpretation without overcomplicating inputs.^[23,35–37]^

Among the compared algorithms, boosting-based methods demonstrated superior discrimination compared with linear classifiers and several conventional machine learning approaches. This advantage is consistent with the ability of modern machine learning models to capture nonlinearities and higher-order interactions in heterogeneous epidemiologic predictors.^[38–41]^ The high negative predictive value observed across models is expected in low-prevalence population evaluation settings,^[42–44]^ whereas modest positive predictive values are typical in such cohorts and depend on prevalence and decision thresholds rather than necessarily indicating model failure.^[45–47]^

We used SHAP to enhance interpretability by providing global explanations for the final model outputs.^[48–51]^ In our model, SHAP highlighted key contributors spanning behavioral exposure (alcohol use), cardiometabolic markers (triglycerides), immune–inflammatory indices (e.g., NLR and absolute lymphocyte count), tobacco exposure (serum cotinine), and environmental exposure biomarkers (e.g., blood lead). The cross-domain nature of these predictors is consistent with CRC being shaped by systemic metabolic–inflammatory states and long-term exposure profiles.^[52–56]^

Inflammation-linked indices such as NLR reflect innate immune activation and have been associated with CRC occurrence, progression, and outcomes in multiple clinical settings.^[57–60]^ Metabolic markers and central adiposity capture insulin resistance, dyslipidemia, and adipose-driven inflammation, which are implicated in colorectal carcinogenesis via endocrine signaling and inflammatory mediators.^[61–64]^ Tobacco exposure biomarkers such as cotinine may index cumulative exposure to carcinogens and immune perturbations that contribute to CRC risk beyond self-reported smoking status.^[26,65,66]^ Heavy metal exposure, including lead, has been linked to oxidative stress, epigenetic modulation, and immune dysregulation, providing a plausible mechanistic basis for associations with cancer susceptibility.^[67–70]^ While our study is predictive rather than causal, the consistency between model-selected predictors and biologically grounded pathways supports plausibility and strengthens confidence in the learned risk signals.^[71–73]^

Prior CRC status classification efforts have often relied on traditional regression-based scores using demographics and lifestyle variables, with modest discrimination and limited ability to capture complex interactions.^[8,74–76]^ More recent machine learning studies, particularly those using boosting methods, have reported improved classification performance through nonlinear modeling and integration of broader feature sets.^[23]^ Our study extends this literature by benchmarking multiple algorithms under a unified framework jointly evaluating discrimination, calibration, decision-analytic utility, and global interpretability using SHAP.^[77]^ Differences between our findings and previous reports may reflect variation in population sampling, CRC definition (self-report vs registry-confirmed), covariate availability, class imbalance handling, and validation strategy.^[78]^ In particular, population-based surveys such as NHANES provide broad generalizability but may differ from hospital-based cohorts in disease spectrum, comorbidity profiles, and biomarker distributions, which can influence both model performance and selected predictors.^[76]^

From an application perspective, the final XGBoost model showed encouraging apparent calibration after post hoc Platt scaling and favorable exploratory decision-analytic performance in the validation cohort, supporting its potential role as an adjunct for population evaluation and clinical triage workflows. However, because probability calibration was fitted on validation-set predictions and then assessed in the same cohort, these probability-based findings require confirmation in an external dataset or a separately reserved calibration sample. Calibration is critical when predicted probabilities are intended to guide actions, and transparent reporting of calibration is emphasized in contemporary prediction model reporting standards.^[79]^ Decision curve analysis further links model outputs to clinical consequences by quantifying net benefit across threshold probabilities.

The accompanying web-based classifier provides an accessible interface for individualized status estimation and illustrates a practical route from model development to dissemination. Online classifier and deployable tools can facilitate real-world uptake, provided that they are accompanied by transparent documentation, input constraints, and ongoing validation.^[80,81]^

Several limitations should be acknowledged. First, CRC status in NHANES may rely on self-report, introducing potential misclassification and attenuation of associations; linkage to registry-confirmed outcomes would strengthen validity in future work. Second, our approach addressed class imbalance using SMOTE and stratified sampling; although widely used, synthetic oversampling can introduce artifacts and may not fully replicate true minority-class structure, highlighting the need for sensitivity analyses using alternative imbalance strategies (e.g., class weights, focal loss, or ensemble balancing).^[82]^ Third, the cross-sectional design limits causal inference and temporality; prospective models with incident CRC outcomes and time-to-event modeling would further enhance clinical relevance.^[76]^ Fourth, generalizability beyond NHANES requires external validation across diverse populations, settings, and measurement platforms, especially when models are intended for broad deployment.^[83]^ Fifth, Platt scaling was fitted post hoc on validation-set predictions and then evaluated in the same cohort; therefore, calibration statistics and decision-curve findings based on calibrated probabilities may be optimistic and should be regarded as exploratory until independently confirmed. Finally, although SHAP improves interpretability, explanation methods can be sensitive to feature correlation and data shifts; robust reporting and stability checks are recommended.^[84]^ Future external validation and structured risk-of-bias appraisal using PROBAST are warranted to support transparent evaluation and potential implementation.^[85]^ Reporting of the present prediction-model study was additionally guided by the TRIPOD + AI statement.^[86]^ A further limitation is survivorship bias inherent to the cross-sectional NHANES design: individuals who died from CRC prior to the survey examination would not be represented in the dataset. The model therefore reflects characteristics of living CRC patients at survey, which may differ from the full spectrum of CRC cases in the population. Standard NHANES complex survey weights could not be applied to our case-control sample because the 1:10 oversampling of CRC cases alters the sampling denominator. Results should therefore be interpreted with this in mind and may not directly reflect weighted national prevalence estimates.

In conclusion, this study presents an interpretable machine learning framework for CRC status classification in a large, population-based dataset, demonstrating strong discrimination. Apparent improvements in probability calibration and decision-analytic utility after post hoc Platt scaling were encouraging but should be regarded as exploratory until confirmed in independent data. These findings motivate further external validation and prospective evaluation, as well as integration of the tool into clinical evaluation, triage, or further-assessment workflows.

## Acknowledgments

The authors thank the participants and investigators of the NHANES for making the data publicly available.

## Author contributions

**Conceptualization:** Jialong Chen.

**Data curation:** Jialong Chen.

**Formal analysis:** Jialong Chen.

**Methodology:** Jialong Chen.

**Software:** Jialong Chen.

**Validation:** Jialong Chen.

**Visualization:** Jialong Chen

**Writing – original draft:** Jialong Chen.

**Writing – review & editing:** Jialong Chen.


