# Supplementary material for: Development and validation of a machine learning model for colorectal cancer status classification using NHANES data: A cross-sectional study
Source: Medicine (Baltimore). 2026 Jun 5;105(23):e49114. doi: 10.1097/MD.0000000000049114 (PMC13246055; doi:10.1097/MD.0000000000049114)
Supplement: Supplementary file 1 [file medi-105-e49114-s001.docx]

**Supplementary Table S1. Candidate predictors, NHANES variable codes, units, and preprocessing notes.**

| **No.** | **Predictor in manuscript** | **NHANES code / source** | **Official variable name** | **Category** | **Type** | **Unit / levels** | **Transformation used** | **Missingness note** | **Brief rationale** |
| --- | --- | --- | --- | --- | --- | --- | --- | --- | --- |
| 1 | Age | RIDAGEYR | Best age in years at screening | Demographics | Continuous | years | None | 0.00% in overall sample | Core demographic predictor; CRC prevalence increases with age. |
| 2 | Sex | RIAGENDR | Gender of the sample person | Demographics | Categorical | Male/Female | Binary encoding | 0.00% in overall sample | Common baseline demographic covariate in CRC studies. |
| 3 | Race/ethnicity | RIDRETH1 | Recode of reported race and ethnicity information | Demographics | Categorical | 5 groups | Categorical encoding | 0.00% in overall sample | Captures population heterogeneity in CRC burden and care pathways. |
| 4 | Education | DMDEDUC2 | Highest grade or level of school completed | Socioeconomic | Categorical | 5 levels | Ordinal/categorical encoding | 0.40% in overall sample | Socioeconomic indicator related to health behaviors and access to care. |
| 5 | Poverty income ratio | INDFMPIR | Poverty income ratio (family income to poverty threshold) | Socioeconomic | Continuous | ratio | None | 9.70% in overall sample | Socioeconomic disadvantage may influence CRC-related exposures and access. |
| 6 | Body mass index | BMXBMI | Body Mass Index | Anthropometry | Continuous | kg/m² | None | 2.10% in overall sample | Adiposity is associated with colorectal carcinogenesis. |
| 7 | Waist circumference | BMXWAIST | Waist Circumference | Anthropometry | Continuous | cm | None | 3.50% in overall sample | Reflects central adiposity and metabolic risk. |
| 8 | Smoking biomarker status | LBXCOT (derived binary) | Cotinine | Lifestyle / exposure | Binary | ng/mL | Binary classification from serum cotinine | 6.80% in overall sample (binary smoking variable in Table 1) | Objective marker of tobacco exposure. |
| 9 | Alcohol use | ALQ101 | Had at least 12 alcoholic drinks in any one year | Lifestyle | Binary | Yes/No | Binary encoding | 0.20% in overall sample (draft based on uploaded table) | Behavioral exposure highlighted by SHAP in the manuscript. |
| 10 | Diabetes | DIQ010 | Ever told by a doctor that participant had diabetes | Comorbidity | Binary | Yes/No | Binary encoding | 0.00% in overall sample | Relevant metabolic comorbidity associated with CRC-related risk patterns. |
| 11 | Hypertension | BPQ020 | Ever told by a doctor that participant had hypertension | Comorbidity | Binary | Yes/No | Binary encoding | 0.40% in overall sample | Common cardiometabolic comorbidity highlighted by SHAP in the manuscript. |
| 12 | Serum cotinine | LBXCOT | Cotinine | Environmental / exposure | Continuous | ng/mL | None | 0.00% in overall sample | Objective tobacco-exposure biomarker. |
| 13 | Blood cadmium | LBXBCD | Cadmium | Environmental / exposure | Continuous | µg/L | None | 0.00% in overall sample | Heavy-metal exposure biomarker. |
| 14 | Blood lead | LBXBPB | Lead | Environmental / exposure | Continuous | µg/dL | None | 0.00% in overall sample | Heavy-metal exposure biomarker highlighted by SHAP in the manuscript. |
| 15 | Blood mercury | LBXTHG | Mercury, total | Environmental / exposure | Continuous | µg/L | None | 0.00% in overall sample | Heavy-metal exposure biomarker. |
| 16 | Neutrophil-to-lymphocyte ratio | Derived from LBXNEPCT and LBXLYPCT | Neutrophil-to-Lymphocyte Ratio | Derived inflammatory index | Continuous | ratio | LBXNEPCT / LBXLYPCT | 0.30% in overall sample | Composite systemic inflammatory marker. |
| 17 | Platelet-to-lymphocyte ratio | Derived from LBXPLTSI and LBXLYPCT | Platelet-to-Lymphocyte Ratio | Derived inflammatory index | Continuous | ratio | LBXPLTSI / LBXLYPCT | 0.30% in overall sample | Composite inflammatory marker used in CRC literature. |
| 18 | Systemic immune-inflammation index | Derived from LBXPLTSI, LBXNEPCT, LBXLYPCT | Systemic Immune-Inflammation Index | Derived inflammatory index | Continuous | index | (LBXPLTSI × LBXNEPCT) / LBXLYPCT | 0.40% in overall sample | Integrated immune-inflammatory index. |
| 19 | C-reactive protein | LBXCRP | C-reactive protein | Inflammation | Continuous | mg/dL | None | 0.30% in overall sample | Inflammatory biomarker. |
| 20 | White blood cell count | LBXWBCSI | White blood cell count | Complete blood count | Continuous | 1000 cells/µL | None | 0.20% in overall sample | Routine hematologic marker of systemic inflammation. |
| 21 | Absolute neutrophil count | LBDNENO | Segmented neutrophils number | Complete blood count | Continuous | 1000 cells/µL | None | 0.30% in overall sample | Absolute innate immune cell count. |
| 22 | Absolute lymphocyte count | LBDLYMNO | Lymphocyte number | Complete blood count | Continuous | 1000 cells/µL | None | 0.30% in overall sample | Adaptive immune cell count highlighted by SHAP in the manuscript. |
| 23 | Platelet count | LBXPLTSI | Platelet count SI | Complete blood count | Continuous | 1000 cells/µL | None | 0.20% in overall sample | Routine platelet marker used to derive PLR and SII. |
| 24 | Fasting plasma glucose | LBXGLU | Fasting Glucose | Biochemistry | Continuous | mg/dL | None | 48.00% in overall sample | Metabolic biomarker. |
| 25 | Glycated hemoglobin (HbA1c) | LBXGH | Glycohemoglobin | Biochemistry | Continuous | % | None | 0.40% in overall sample | Longer-term glycemic control marker. |
| 26 | Total cholesterol | LBXTC | Total cholesterol | Biochemistry | Continuous | mg/dL | None | 3.40% in overall sample | Lipid marker included in the uploaded baseline table. |
| 27 | Triglycerides | LBXTR | Triglyceride | Biochemistry | Continuous | mg/dL | None | 48.00% in overall sample | Metabolic marker highlighted by SHAP in the manuscript. |
| 28 | HDL cholesterol | LBDHDD | Direct HDL-Cholesterol | Biochemistry | Continuous | mg/dL | None | 3.40% in overall sample | Lipid marker commonly used in metabolic profiling. |

Abbreviations: BMI, body mass index; CRC, colorectal cancer; HDL, high-density lipoprotein; HbA1c, glycated hemoglobin; NLR, neutrophil-to-lymphocyte ratio; PLR, platelet-to-lymphocyte ratio; SII, systemic immune-inflammation index.
